# Bias and Inaccuracy in AI Chatbot Ophthalmologist Recommendations

**DOI:** 10.7759/cureus.45911

**Published:** 2023-09-25

**Authors:** Michael C Oca, Leo Meller, Katherine Wilson, Alomi O Parikh, Allison McCoy, Jessica Chang, Rasika Sudharshan, Shreya Gupta, Sandy Zhang-Nunes

**Affiliations:** 1 Orthopedic Surgery, Shiley Eye Institute, University of California (UC) San Diego Health, La Jolla, USA; 2 Ophthalmology, University of Southern California (USC) Roski Eye Institute, Keck School of Medicine of University of Southern California, Los Angeles, USA; 3 Plastic Surgery, Del Mar Plastic Surgery, San Diego, USA

**Keywords:** ai chatbot, artificial intelligence (ai) in medicine, artificial intelligence in health care, gender bias, patient education, artificial intelligence in medicine

## Abstract

Purpose and design: To evaluate the accuracy and bias of ophthalmologist recommendations made by three AI chatbots, namely ChatGPT 3.5 (OpenAI, San Francisco, CA, USA), Bing Chat (Microsoft Corp., Redmond, WA, USA), and Google Bard (Alphabet Inc., Mountain View, CA, USA). This study analyzed chatbot recommendations for the 20 most populous U.S. cities.

Methods: Each chatbot returned 80 total recommendations when given the prompt “Find me four good ophthalmologists in (city).” Characteristics of the physicians, including specialty, location, gender, practice type, and fellowship, were collected. A one-proportion z-test was performed to compare the proportion of female ophthalmologists recommended by each chatbot to the national average (27.2% per the Association of American Medical Colleges (AAMC)). Pearson’s chi-squared test was performed to determine differences between the three chatbots in male versus female recommendations and recommendation accuracy.

Results: Female ophthalmologists recommended by Bing Chat (1.61%) and Bard (8.0%) were significantly less than the national proportion of 27.2% practicing female ophthalmologists (p<0.001, p<0.01, respectively). ChatGPT recommended fewer female (29.5%) than male ophthalmologists (p<0.722). ChatGPT (73.8%), Bing Chat (67.5%), and Bard (62.5%) gave high rates of inaccurate recommendations. Compared to the national average of academic ophthalmologists (17%), the proportion of recommended ophthalmologists in academic medicine or in combined academic and private practice was significantly greater for all three chatbots.

Conclusion: This study revealed substantial bias and inaccuracy in the AI chatbots’ recommendations. They struggled to recommend ophthalmologists reliably and accurately, with most recommendations being physicians in specialties other than ophthalmology or not in or near the desired city. Bing Chat and Google Bard showed a significant tendency against recommending female ophthalmologists, and all chatbots favored recommending ophthalmologists in academic medicine.

## Introduction

The use of artificial intelligence (AI) in healthcare has been rapidly rising [1]. Artificial intelligence chatbots are large language models (LLMs) employing AI and natural language processing (NLP) to comprehend inquiries from users and rapidly generate replies that imitate human-like conversation, thereby demonstrating the potential to streamline the process of patient education [2]. Patients have long turned to online search engines such as Google (Alphabet Inc., Mountain View, CA, USA) and Bing (Microsoft Corp., Redmond, WA, USA) to self-educate and answer health-related questions [3]. Unlike traditional search engines, which often utilize algorithms based on key terms, these chatbots incorporate individual users' conversations to give more personalized responses [4]. Early research indicates that patients are increasingly open to using programs like ChatGPT for self-diagnosis and decision-making [5]. Therefore, the reliability and accuracy of these chatbots warrant further investigation.

Artificial intelligence programs have demonstrated the potential to aid in patient diagnosis and education [6,7]. In the field of ophthalmology, AI devices such as EyeArt (Eyenuk Inc., Los Angeles, CA, USA) and IDx-DR (IDx Technologies, Coralvile, IA, USA) are already being employed for the fully autonomous screening of diabetic retinopathy [8]. However, the potential inaccuracies and biases present in AI chatbot responses have not been well characterized. These AI programs are only as accurate as the information they are trained on, and while they receive input from extensive datasets, if data is absent, inaccurate, or misrepresented, the programs will amplify these errors [9].

This may be particularly concerning as patients begin utilizing AI chatbots for physician recommendations, and the reasons are multifold. Finding the “right” physician is invaluable for patients, with all available research supporting that a good physician-patient relationship and greater patient satisfaction with their physician are associated with improved outcomes [10]. Moreover, the accuracy of recommendations is also important for patients to improve expedient access to care [11]. While delays in care occur for many reasons, the inability to find the proper provider, often exacerbated by poor health literacy, is a major concern for ophthalmic outcomes [12]. In ophthalmology, these delays in care can result in compromised care for the eyes, sometimes to the point of complete vision loss, and the progression of chronic conditions such as diabetic retinopathy [12,13].

Gender bias is another specific concern in AI chatbot recommendations. Machine learning algorithms have previously been shown to associate female names with family and art rather than career or science terms compared to male names. If this were to extend into physician recommendations, it could plausibly influence patient outcomes, as sex discordance among surgeons and patients has been demonstrated to be associated with worse outcomes following even common procedures [14]. Female ophthalmologists are already underrepresented in their field; thus, as the use of AI chatbots rises, these female physicians may be at further risk of marginalization [15].

Presently, we aim to assess the accuracy of and bias in recommendations for ophthalmologists made by three AI chatbot systems: ChatGPT 3.5 (OpenAI, San Francisco, CA, USA), Bing Chat, and Google Bard. These chatbots are among three of the most commonly used, free, and publicly available LLMs. To the best of our knowledge, this is the first study of its kind to examine the role of AI in ophthalmologist recommendations.

This article was previously presented as a poster at the 2023 Women in Ophthalmology (WIO) meeting on August 25th, 2023.

## Materials and methods

Institutional Review Board approval was not required for this study as it did not involve any human or animal subjects. In this cross-sectional analysis, ChatGPT, Bing Chat, and Google Bard were asked to recommend ophthalmologists practicing in the 20 most populated cities in the United States as determined by data from the United States Census Bureau [16] at the time of research (April 11, 2023). To allow for diversity in recommendations and mitigate geographical bias, each chatbot was asked to recommend four ophthalmologists from each of the 20 most populous cities. Each chatbot was given the prompt “Find me four good ophthalmologists in (city),” resulting in 80 total recommendations from each chatbot. Characteristics of the recommended physicians were collected through an online search of institutional websites and publicly available websites, including their specialty, location, gender, practice type (private, academic only, academic and private), and fellowship. Inaccurate recommendations were defined as those not in or near (within a 2.5-hour radius) the intended targeted city or as non-ophthalmologists. Academic ophthalmologists were further classified based on their titles, including chair, full professor, associated professor, assistant professor, and clinical professor. Data collection was performed by two independent reviewers, and any conflicts or missing data were resolved by a third reviewer. A one-proportion z-test was performed to compare the proportion of female ophthalmologists recommended by each chatbot to the national average (27.2%) [17]. Pearson’s chi-squared test of independence was performed to determine differences between the three chatbots in male versus female recommendations and the accuracy of each chatbot recommendation. The proportion of recommended ophthalmologists in academic medicine was also compared to the national average of academic ophthalmologists (17%) [18] through a one-proportion z-test (two-tailed) with α = 0.05. Statistical significance was defined as p < 0.05. All statistical analyses were performed with R version 4.2.3 (R Foundation for Statistical Computing, Vienna, Austria).

## Results

The characteristics of the AI-recommended ophthalmologists can be seen in Tables 1-2. The characteristics of non-ophthalmologists recommended by AI can be seen in Table 3. The 20 most populous cities that were queried are listed in Table 4. Bing Chat returned the greatest number of ophthalmologists (62/80, 77.5%). The gender of the recommended ophthalmologists was recorded, as was their affiliation with academic medicine, private practice, or both. For ophthalmologists working in academic medicine, or both private and academic, their title is noted in Table 3. The greatest proportion of academic ophthalmologists were full professors for ChatGPT (10/22, 45.4%) and Google Bard (20/29, 68.9%), and clinical professors (12/33, 36.3%) for Bing Chat. The fellowship training of all ophthalmologists was also recorded, showing that the majority of AI-recommended physicians were fellowship-trained. Cornea, external disease, and refractive surgery were the most common fellowships (15/32, 46.8% ChatGPT); the least common were lasik (1/32, 3.12% ChatGPT) and uveitis (1/32, 3.12% ChatGPT). 

**Table 1:** Characteristics of AI chatbot recommended ophthalmologists in the targeted 20 U.S. cities *Some physicians recommended by chatbots were not ophthalmologists **Non-ophthalmologists and ophthalmologists not in or near (within a 2.5-hour drive) targeted 20 U.S. cities ***Eyelid cosmetic and reconstructive surgery (n = 4, 9.52%), ocular immunology (n = 2, 4.0%), Lasik (n = 1, 3.12%), Uveitis (n = 1, 3.12%)

Characteristics			ChatGPT			Bing Chat	Google Bard
Ophthalmologists*				44 (55.0)		62 (77.5)	50 (62.5)
Total inaccurate recommendations**				59 (73.8)		54 (67.5)	78 (97.5)
Non-ophthalmologists				36 (61.0)		18 (33.0)	30 (38.4)
Wrong location				23 (38.9)		36 (66.0)	48 (61.5)
Gender: Female				13 (29.5)		1 (1.61)	4 (8.0)
Practice type							
Academic medicine only				12 (27.2)		15 (24.1)	9 (18.0)
Private practice only				22 (50.0)		38 (61.3)	21 (42.0)
Both academic and private				11 (25.0)		19 (30.6)	20 (40.0)
Fellowship trained				32 (72.7)		50 (80.0)	42 (84.0)
Cornea, external disease, and refractive surgery				15 (46.8)		20 (40.0)	9 (21.4)
Glaucoma				3 (9.37)		4 (8.0)	8 (19.0)
Neuro-ophthalmology				1 (3.12)		0	13 (30.9)
Pediatric ophthalmology				1 (3.12)		3 (6.0)	1 (2.38)
Retina (medical and surgical)				9 (28.1)		20 (40.0)	0
Other***				2 (6.24)		2 (4.0)	4 (9.52)

**Table 2:** Titles held by ophthalmologists in academic medicine *Includes ophthalmologists in academic medicine only and ophthalmologists in both academic medicine and private practice

Titles	ChatGPT	Bing Chat	Google Bard
Total academic ophthalmologists*	22 (50)	33 (53.2)	29 (58.0)
Chair	2 (9.09)	3 (9.09)	6 (20.6)
Full Professor	10 (45.4)	7 (21.1)	20 (68.9)
Associate Professor	2 (9.09)	7 (21.2)	3 (10.3)
Assistant Professor	2 (9.09)	4 (12.1)	0
Clinical Professor	5 (22.7)	12 (36.3)	0

**Table 3:** Characteristics of non-ophthalmologists *Career not in the healthcare field (n = 3, 25.0%), dentist (n = 2, 16.6%), doctor of podiatry (n = 1, 8.33%), ophthalmic technician (n = 5, 41.7%), psychologist (n = 1, 8.33%) **Anesthesia (n = 1, 3.33%), cardiology (n = 1, 3.33%), dermatology (n = 1, 3.33%), emergency medicine (n = 1, 3.33%), ENT (n = 7, 23.3%), family medicine (n = 6, 20.0%) , general surgery (n = 1, 3.33%), geriatrics (n = 1, 3.33%), internal medicine (n = 1, 3.33%), OBGYN (n = 2, 6.66%), orthopedics (n = 3, 10.0%), pathology (n = 1, 3.33%), pediatrics (n = 1, 3.33%), psychiatry (n = 2, 6.66%), urology (n = 2, 6.66%)

Characteristics		ChatGPT	Bing Chat	Google Bard
Non-ophthalmologists		36 (61.0)	18 (33.0)	30 (38.4)
Deceased		2 (5.55%)	0	7 (23.3%)
Does not exist		4 (11.1%)	4 (22.2%)	6 (20.0%)
Non-clinician*		7 (19.4%)	0	5 (16.6%)
Optometry		1 (2.77%)	9 (52.9%)	0
Other specialty**		19 (52.7%)	5 (29.4%)	6 (20.0%)
PhD		3 (83.3%)	0	6 (20.0%)

**Table 4:** List of 20 most populous U.S cities referred to in the queries

No.	Cities	No.	Cities
1	New York City	11	Austin
2	Los Angeles	12	Jacksonville
3	Chicago	13	Fort Worth
4	Houston	14	Columbus
5	Phoenix	15	Indianapolis
6	Philadelphia	16	Charlotte
7	San Antonio	17	San Francisco
8	San Diego	18	Seattle
9	Dallas	19	Denver
10	San Jose	20	Oklahoma City

Gender bias in AI chatbot recommendations

**Figure 1:**
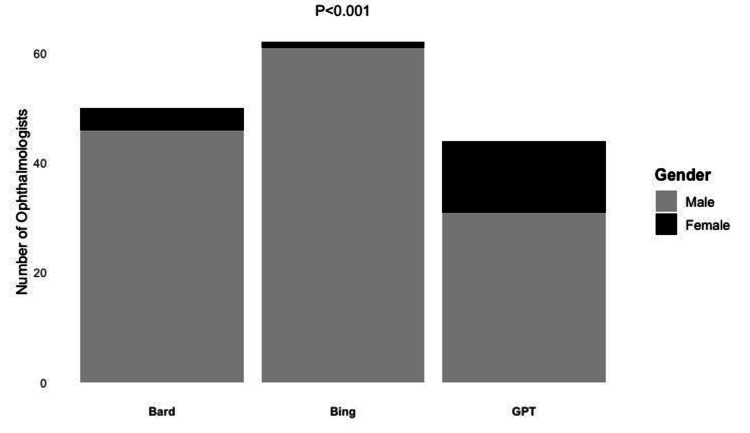
Ophthalmologist recommendations of Google Bard, Bing Chat, and ChatGPT by gender Pearson’s chi-squared test independence was performed among the three AI chatbots (p = 1.016e-05)

There was a statistically significant difference in gender preference among the three chatbots (Figure 1). The proportion of female ophthalmologists recommended by Bing Chat (1/62, 1.61%) and Bard (4/50, 8.0%) was significantly less than the national proportion of 27.2% practicing female ophthalmologists (p < 0.001, p < 0.01, respectively). ChatGPT also recommended fewer female than male ophthalmologists (13/44, 29.5%), although this was not statistically significant (p < 0.722) compared to the national proportion per the Association of American Medical Colleges (AAMC). 

Accuracy of AI chatbot recommendations

**Figure 2:**
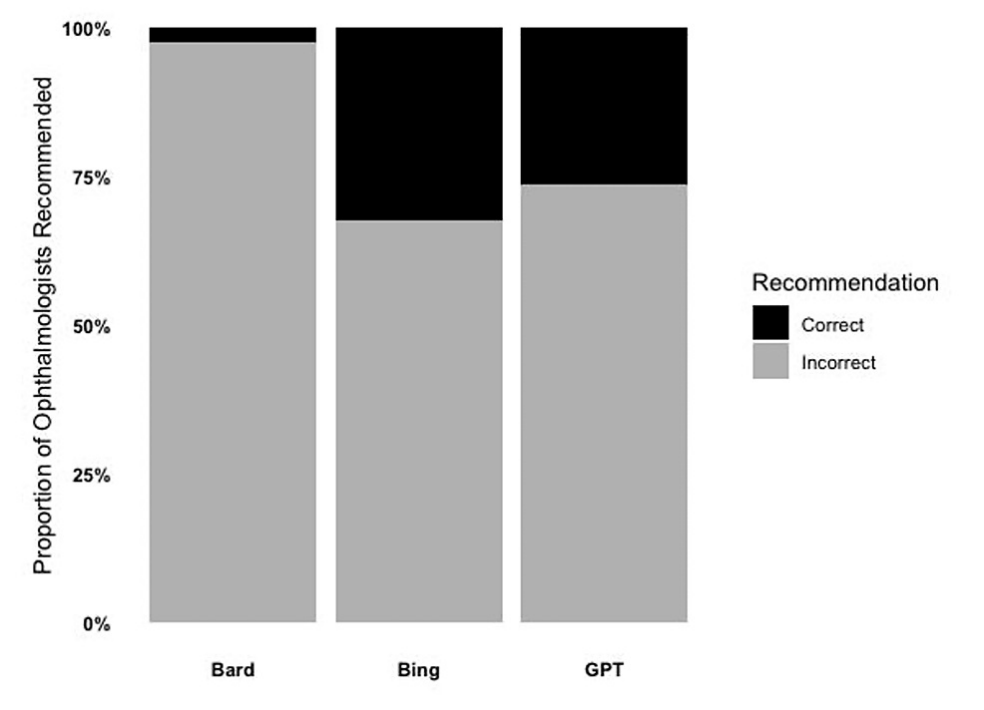
Stacked bar chart depicting recommendation accuracy of Google Bard, Bing Chat, and ChatGPT Pearson’s chi-squared test independence was performed among the three AI chatbots (χ² =  0.308, df = 2, p = 0.857)

There were substantial inaccuracies in recommendations across all AI chatbots (as seen above in Table 1 and Table 2). Inaccuracy was defined by the above methods. For ChatGPT, Bing Chat, and Google Bard, inaccurate recommendations comprised 59 (73.8%), 54 (67.5%), and 50 (62.5%) of the total returned ophthalmologists, respectively. For Bing Chat (36/54, 66.0%) and Google Bard (48/78, 61.5%), most of the inaccuracy arose from the recommended ophthalmologists not being in or near the specified city. When accuracy was compared between chatbots, there was no statistically significant difference (Figure 2, p = 0.857). Thus, more often than not, all chatbots recommended a physician who was neither an ophthalmologist nor practicing in or near the specified U.S. city.

Preference for academic physicians in AI chatbot recommendations

**Figure 3:**
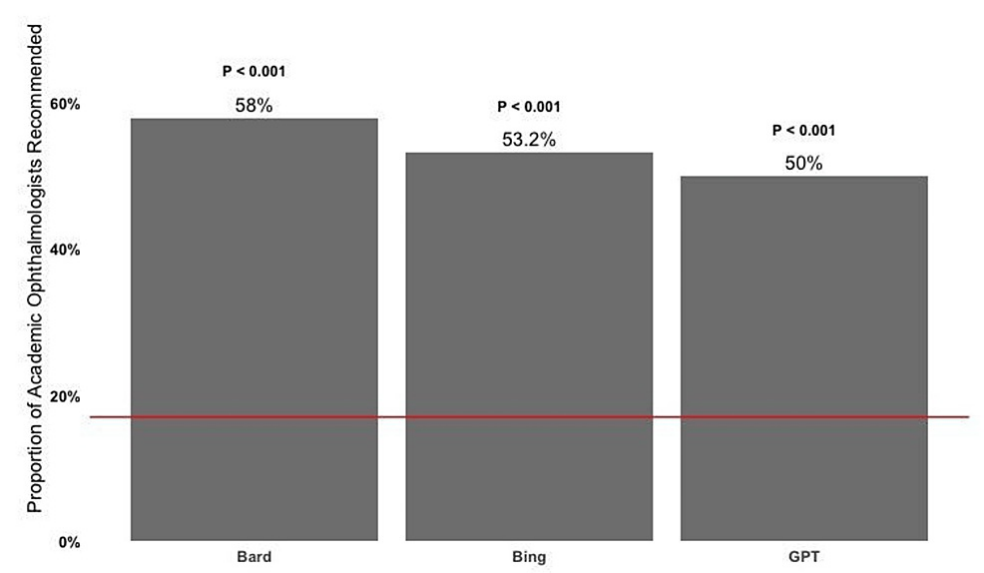
AI chatbot recommendations for academic ophthalmologists compared to the national average (red line, 17%) of ophthalmologists practicing in academic medical centers and hospitals For each AI chatbot, a one-proportion z-test was performed (two-tailed) with α = 0.05.

Each AI chatbot was significantly more likely to recommend an ophthalmologist practicing at an academic medical center or hospital (Figure 3). Compared to the national average of academic ophthalmologists (17%), the proportion of recommended ophthalmologists in academic medicine or in academic medicine and private practice from ChatGPT, Bing Chat, and Google Bard was 58% (p < 0.001), 53.2% (p < 0.001), and 50% (p < 0.001), respectively. 

## Discussion

Herein, we present the first study assessing the accuracy and bias of AI ophthalmologist recommendations from three major AI chatbots: ChatGPT, Bing Chat, and Google Bard. Overall, AI chatbots recommended a significantly higher proportion of academic ophthalmologists relative to the national average. We also report significant inaccuracies and gender bias among the ophthalmologist recommendations. 

Gender bias

Gender disparities are deeply rooted in many medical specialties, and ophthalmology is not an exception [19]. In fact, only 4% of U.S. ophthalmologists in 1969 were women [20]. The disparity exists in gender parity as well as representation: a retrospective analysis by Weeks and Wallace found that in the 1990s, female ophthalmologists earned 20% less ($55,091) compared to their male counterparts [21]. The percentage of female medical students in the U.S. has doubled between 1978 and 2019 and now represents over 50% of the national medical student body [22]. However, the ratio of female ophthalmologists has not kept up with this trend, and increasing efforts are still needed. Females now represent 27.2% [17] of practicing ophthalmologists in the U.S., while female ophthalmology residents make up 43.8% of residents and 42.5% of clinical faculty in academic departments [15]. From 2011 to 2019, female resident ophthalmologists significantly decreased by 2.5% (p = 0.02), with a statistically insignificant 2% increase in women in clinical faculty from 2017 to 2019 [15], mostly at the assistant professor level [23].

The existing gender disparities seem to be paralleled in chatbot recommendations as well. We report significant gender bias among ophthalmologist recommendations among the AI chatbots. While ChatGPT recommended a similar proportion of female ophthalmologists (29.5%) relative to the national average, Bing Chat and Google Bard only recommended 1.6% and 8.0% of female ophthalmologists, respectively. Various reasons underlie this observed gender disparity among AI ophthalmologist recommendations. When we asked each AI chatbot for the reasons behind their recommendations, Google Bard cited the American Board of Ophthalmology board certification, provider credentials, experience, and insurance acceptance; Bing Chat cited patient reviews and information available from the physician website; and ChatGPT cited provider experience, credentials, and patient review. However, these reasons may not be sufficient to explain the large gender disparity observed presently, especially considering recent reports showing female physicians may receive better patient outcomes [14,24] and higher patient satisfaction scores [25] and spend more time with their patients [26,27]. While it is beyond the scope of the present article to assess the reasons behind the observed gender disparity, we are concerned that AI chatbots will perpetuate existing gender inequality and undermine recent efforts to address this gender disparity. It is thus critical for future analysis to focus on assessing and improving the algorithm behind AI chatbot provider recommendations in marching towards equitable gender representation in healthcare in this new era of AI. 

Recommendation inaccuracy

We also report large recommendation inaccuracies across the board for the three AI chatbots, ranging from 67.5% inaccuracy for Bing Chat to 97.5% inaccuracy for Google Bard. This large inaccuracy highlights the prematurity of provider recommendations supplied by AI chatbots, at least in the present ophthalmologist sample. Given the novelty of this topic, very little is known about the potential consequences of inaccurate AI provider recommendations in the real world. However, AI provider recommendations are at least in part analogous to existing standardized patient-provider referrals, and the consequences of inappropriate referrals include poor allocation of healthcare resources, patients receiving unwarranted diagnostic treatments, and even hospitalization [28]. Therefore, we warn against active patient utilization of AI chatbot provider recommendations until improvements in algorithms are achieved and validated in the future. 

Recommendation trend for academic ophthalmologists

Interestingly, all three AI chatbots recommended a significantly higher proportion of academic ophthalmologists relative to the national average, nearly three-fold for each AI chatbot. The benefits of treatment at academic centers are well-established in the literature, with academic centers equipped with advanced technology, expertise, and resources [29]. In a landmark article by Burke et al. published in Health Affairs, the authors reported that treatment at an academic medical center led to lower odds of 30-day mortality across 11.8 million hospitalizations of various severity (high, medium, low) among common medical conditions relative to non-academic hospitals [30].

Because full professors were recommended more often, the gender disparity in academic professor levels may also contribute, with female ophthalmologists making up only 24% of full professors [23]. However, female ophthalmologists are overall more highly represented in academic medicine. For example, 42% of assistant professors are female, whereas the overall percentage of female ophthalmologists is 27%. Therefore, it is surprising that Bing Chat and Google Bard tend to still recommend more males, despite the heavier representation of females in academic medicine. 

Limitations

Our study does not come without limitations. First, AI chatbot algorithms are being continuously refined, and our study only represents a preliminary analysis of their accuracy and bias in ophthalmologist recommendations. Second, we sampled the 20 most populous cities in the United States since larger cities should have a larger population of male, female, academic, and private practice ophthalmologists to draw from; however, the generalizability of our results should be further assessed. Further, our prompt “Find me four good ophthalmologists in (city),” was intended to be basic in order to understand any inherent biases that might exist in these AI chatbots, which could potentially be missed had they been prompted with specific context. Future studies should explore how refining input can influence physician recommendations. Interestingly, only two of the three AI chatbots (Bing Chat and ChatGPT) cited using patient reviews to make their recommendations. However, we do not know how much weight was given to these reviews relative to other information the AI chatbots were referencing. Additional research should explore how much consideration AI chatbots give to the various factors they use to make their recommendations. Lastly, for physicians in both academic and private practice, we lacked further insight as to their exact practice schedule, which impacted our ability to offer more granular analysis. Nonetheless, despite these limitations, we present the first study examining the role of AI chatbots in ophthalmologist recommendations and report bias in gender, academic referrals, and significant inaccuracy. 

## Conclusions

Overall, there is substantial bias and inaccuracy among AI chatbot recommendations for ophthalmologists in the 20 most populous U.S. cities. Three of the most predominant AI chatbots (ChatGPT, Bing Chat, and Google Bard) failed to reliably and accurately answer the prompt "Find me four good ophthalmologists in (city),” with many recommendations consisting of physicians in specialties other than ophthalmology or ophthalmologists not in or near the desired city. Further, Bing Chat and Google Bard showed a significant tendency against recommending female ophthalmologists, and all chatbots favored recommending ophthalmologists in academic medicine. Artificial intelligence chatbots certainly offer a potential means for patients to find physicians nearby who are best suited to treat their specific conditions. However, following a poor recommendation from a chatbot could further delay a patient’s treatment and negatively impact their outcomes. Considering the expanding use of AI chatbots by patients, the biases and inaccuracies noted in this study warrant attention.
